# Identification, Cloning, and Characterization of Two Acupuncture-Injury-Inducing Promoters in Rice

**DOI:** 10.3390/ijms251910564

**Published:** 2024-09-30

**Authors:** Jianyu Wang, Zengfeng Ma, Dong Fu, Yan Wu, Zaihui Zhou, Changyan Li, Junhao Shen

**Affiliations:** 1 School of Life Sciences, Hubei University, Hubei Collaborative Innovation Center for Green Transformation of Bio-Resources, Wuhan 430062, China; 202221107012051@stu.hubu.edu.cn (J.W.); fudong@hubu.edu.cn (D.F.); 2 Hubei Key Laboratory of Food Crop Germplasm and Genetic Improvement, Laboratory of Crop Molecular Breeding, Food Crops Institute, Hubei Academy of Agricultural Sciences, Ministry of Agriculture and Rural Affairs, Wuhan 430064, China; yanwu@hbaas.com; 3 Guangxi Key Laboratory of Rice Genetics and Breeding, State Key Laboratory for Conservation and Utilization of Subtropical Agro-Bioresources, Rice Research Institute, Guangxi Academy of Agricultural Sciences, Nanning 530007, China; mazengfeng@gxaas.net; 4 College of Life Science and Technology, Huazhong Agricultural University, Wuhan 430070, China; zaihuizhou@mail.hzau.edu.cn

**Keywords:** rice, transcriptome analysis, inducible promoters, transgenic crops, insect resistance

## Abstract

As an important global food crop, rice is damaged by a variety of piercing–sucking pests. Identifying a broad-spectrum promoter induced by the physical signal of sucking pests and applying it to transgenic breeding to mitigate the damage caused by different sucking pests will significantly improve the efficiency of our breeding. This study compared the transcriptome changes in two rice varieties under needle-wounding stress to investigate their differential responses to mechanical damage. The results showed that the insect-susceptible variety TN1 exhibited more differentially expressed genes (DEGs) and greater changes in expression levels after needle treatment, indicating a more active internal gene regulatory network. GO and KEGG enrichment analysis further revealed that TN1 not only exhibited changes in genes related to the extracellular environment, but also mobilized more genes associated with stress response and defense. By screening the differentially expressed genes, we identified two promoters (P1 and P2) with inducible expression characteristics in both the resistant and susceptible rice varieties. These promoters were able to effectively drive the expression of the insect resistance gene OsLecRK1* and enhance the resistance of transgenic plants against the brown planthopper. This study provides promoter resources for the development of insect-resistant transgenic crops.

## 1. Introduction

Piercing–sucking mouthpart pests are insects that feed on plant sap by piercing and sucking. They are widely present in various crops and insert their stylets into plant tissues to extract nutrients, which severely affects the growth and development of the crops. The main piercing–sucking mouthpart pests include aphids, whiteflies, and planthoppers. During the piercing process, they can also transmit certain plant viral diseases. The excretions of piercing–sucking mouthpart pests on plant leaves contain large amounts of sugars, which promote mold growth on plant surfaces, obstructing green tissues and affecting photosynthesis. Piercing–sucking insects possess needle-like structures that extract food from the phloem of plants. Some insects, such as leafhoppers, planthoppers, and stink bugs, use their stylets to directly penetrate the plant’s epidermal cells and reach the sieve tube tissue [1,2]. Others, such as aphids, whiteflies, and psyllids, maneuver their stylets between plant epidermal cells to eventually reach the sieve tube tissue [3,4].

Plants and herbivorous insects have developed complex defense and counter-defense mechanisms through long-term co-evolution. To cope with herbivorous insect feeding, plants have evolved both constitutive and inducible defense strategies. In response, herbivorous insects have developed sophisticated counter-defense mechanisms to utilize and adapt to their host plants. Herbivorous insects can use effectors in their saliva to interfere with or suppress host plant defense responses, rely on detoxification enzyme systems to metabolize toxic substances derived from plants, indirectly suppress plant insect defenses by carrying symbiotic microorganisms, acquire functional genes through horizontal gene transfer from bacteria and plants to enhance their fitness, and use or manipulate plant volatiles to counteract host plant defenses. For example, piercing–sucking insects significantly increase the accumulation of defense-related proteins and the synthesis of defense-related chemicals (such as lignin, cellulose, alkaloids, callose, phenolics, and silica particles), thereby strengthening the plant cell wall and effectively blocking the feeding of piercing–sucking insects [5,6,7]. Genes such as phenylalanine ammonia-lyase (*OsPALs*), GA receptor gene *OsGID1*, *OsWRKY89*, *BPH6*, *BPH30*, and *BPH40* mediate cell wall fortification and lignin production, enhancing rice resistance to planthoppers [8,9,10,11]. Callose, a glucan polymer, is synthesized by callose synthase genes in various tissues, each performing different functions. However, only callose in sieve pores is associated with the blockage of sieve tube tissues [12]. The BPH14 gene effectively promotes the deposition of callose in sieve tube tissues, significantly enhancing rice resistance to planthoppers. In summary, most of the defense responses mediated by plant disease and insect resistance genes are inducible defenses. Inducible defenses refer to a series of structural and inducible defense responses triggered when plants are affected by external environmental factors or other biological attacks. These defenses involve changing the plant’s physiological and biochemical states, activating multiple molecular signaling pathways, regulating the expression of defense-related genes, and accelerating the accumulation of defensive substances [13]. As research on the cloning of insect resistance genes and their molecular mechanisms continues to deepen, the molecular mechanisms by which defense-related genes function are gradually becoming clearer [14]. Currently, the main types of cloned insect resistance genes in plants encode proteins such as lectin-like receptor kinases (LecRKs) and nucleotide-binding site-leucine-rich repeats (NBS-LRRs) [15]. Inducible defense responses are rapid, efficient, and effective, playing a crucial role in plant defense against insect damage.

Overall, plant defense responses to piercing–sucking mouthpart pests can be divided into two main types: one is the broad-spectrum plant response to the physical damage caused by insect piercing [16,17]; the other is the specific recognition of elicitor compounds in the saliva of certain piercing–sucking pests, which triggers specific induced defense responses [18,19]. Therefore, theoretically, the response of the same crop to the physical damage signals of different piercing–sucking insects should be similar, but the induced defense responses to different pests and elicitors will vary. If we could identify a broad-spectrum promoter that is induced by the physical signals of piercing–sucking pests and apply it to transgenic breeding, we could address the damage caused by these pests more effectively, greatly enhancing breeding efficiency.

Rice, as a major food crop in China, is vulnerable to various piercing–sucking pests. Common pests include the brown planthopper, white-backed planthopper, leafhoppers, and rice thrips [20,21,22]. The brown planthopper is a migratory pest widely distributed in Asia, causing particularly severe damage in southern China’s rice-growing regions. The white-backed planthopper primarily affects areas south of the Yangtze River in China, posing significant threats to rice crops. Leafhoppers and rice thrips feed on rice in both adult and larval stages, hindering rice growth.

In transgenic rice breeding for insect resistance, specific inducible promoters are a class of promoters that can induce gene expression under specific environmental conditions. In transgenic breeding, specific inducible promoters can be used to regulate the expression of insect-resistant genes, allowing them to function when pests attack, thereby improving crop resistance to insects [23,24,25,26]. Research has successfully utilized specific inducible promoters to regulate the expression of insect-resistant genes, resulting in the development of insect-resistant rice varieties. These studies provide new ideas and methods for insect-resistant transgenic breeding.

To explore resistance gene resources, many researchers have used transcriptome analysis techniques to study the changes in gene expression in rice during piercing–sucking pest attacks. By analyzing differentially expressed genes in rice under various pest infestations, they have identified a set of genes with potential insect-resistant functions [27]. Further investigation into the promoters of these genes aims to identify broad-spectrum inducible promoters triggered by piercing–sucking pests, offering new research directions for developing insect-resistant transgenic rice. Additionally, combining the specific inducible promoters mentioned in the previous paragraph, there is potential to achieve precise expression of insect-resistant genes under specific environmental conditions, thereby enhancing crop resistance to insects.

In summary, by describing the types of piercing–sucking mouthpart pests and damages caused by, as well as distinguishing them from chewing pests, compiling resources of resistance genes against piercing–sucking pests, exploring the role of specific inducible promoters in transgenic breeding, and seeking broad-spectrum inducible promoters through transcriptome analysis, a theoretical basis and technical support have been provided for the cultivation of insect-resistant transgenic rice. It is worth noting that, so far, we have not found similar studies in rice or the application of promoters with broad-spectrum responses to piercing–sucking pests. Looking ahead, with the continuous development of scientific technologies, there is potential to achieve more efficient and environmentally friendly crop insect resistance breeding, thereby providing sustained support and progress for agricultural production.

## 2. Results

### 2.1. Transcriptome Analysis of RH and TN1 after Needle Pricking Treatment

**Figure 1:**
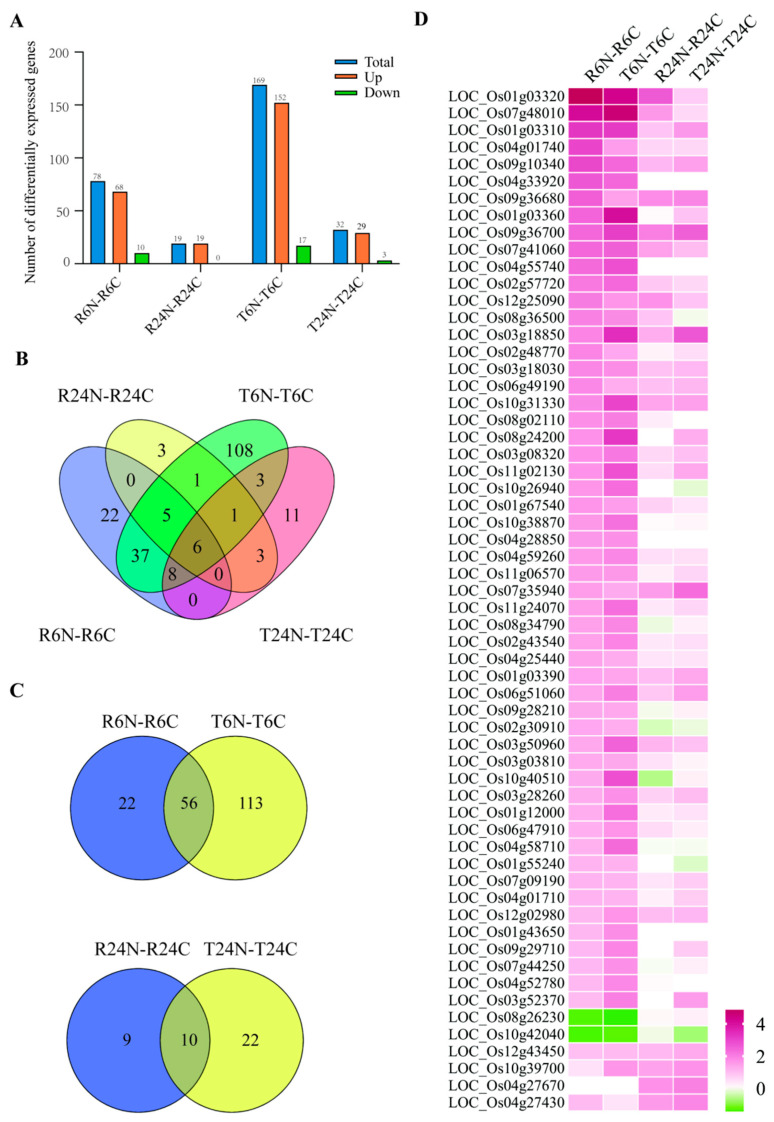
Analysis of differentially expressed genes in RH and TN1 after needle pricking treatment. (**A**): Number of upregulated and downregulated differentially expressed genes. (**B**): Venn diagram of differentially expressed genes among the four treatment groups. (**C**): Venn diagram of differentially expressed genes at 6 and 24 h after needle pricking. (**D**): Heatmap of the differentially expressed genes shared between TN1 and RH.

Among the four treatment groups (R6N-R6C, R24N-R24C, T6N-T6C, and T24N-T24C), there were 78, 19, 169, and 32 differentially expressed genes (DEGs) (fold change ≥ 2), respectively. At 6 h after needle pricking, RH and TN1 had 68 and 29 upregulated genes, and 10 and 17 downregulated genes, respectively. At 24 h after needle pricking, the number of differentially expressed genes decreased significantly, with RH and TN1 having 19 and 29 upregulated genes, respectively, and RH having no downregulated genes, while TN1 had 3 downregulated genes (Figure 1A). Since the needle pricking damage to rice is a one-time event and not a continuous stress, most of the initial differentially expressed genes may no longer be induced by the damage signal at 24 h after treatment.

A Venn diagram showed that the four treatment groups actually had only 208 differentially expressed genes in total (Figure 1B). Furthermore, in the comparisons between the two varieties at the same time point, R6N/T6N and R24N/T24N shared 56 and 10 differentially expressed genes, respectively. However, at 6 h, RH and TN1 had 22 and 113 unique differentially expressed genes, respectively, while at 24 h, RH and TN1 had 9 and 22 unique differentially expressed genes, respectively (Figure 1C). We then selected the differentially expressed genes shared between the two varieties and combined the results from 6 and 24 h, finding 60 differentially expressed genes that were induced by needle pricking in both RH and TN1. Among all of the differentially expressed genes, the majority were upregulated, and the changes in TN1 seemed to be more pronounced than in RH (Figure 1D). Taken together, these results suggest that TN1 may be more sensitive to needle pricking injury and have a more active internal gene regulatory network compared to RH.

### 2.2. GO Enrichment Analysis of DEGs in the Four Treatment Groups

**Figure 2:**
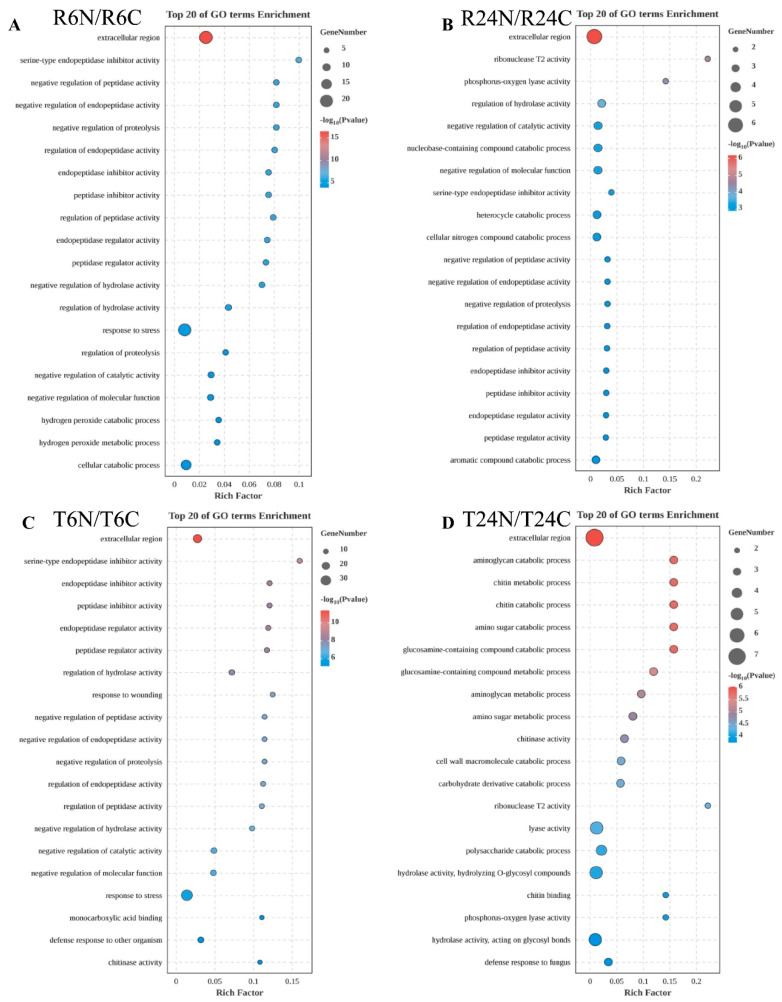
GO enrichment analysis of differentially expressed genes in the four treatment groups. (**A**): GO enrichment analysis of differentially expressed genes in R6N-R6C. (**B**): GO enrichment analysis of differentially expressed genes in R24N-R24C. (**C**): GO enrichment analysis of differentially expressed genes in T6N-T6C. (**D**): GO enrichment analysis of differentially expressed genes in T24N-T24C.

In the comparison between R6N and R6C, the most significantly enriched GO term was the extracellular region (GO:0005576), with a high enrichment factor, indicating that under this condition, a large number of genes related to biological processes in the extracellular region were significantly differentially expressed. Other significantly enriched GO terms included serine-type endopeptidase inhibitor activity (GO:0004867) and negative regulation of peptidase activity (GO:0010466), which are related to protein degradation and regulation (Figure 2A). In the comparison between R24N and R24C, the extracellular region was still the most significantly enriched GO term. Additionally, ribonuclease T2 activity (GO:0004540) and phosphorus–oxygen lyase activity (GO:0016849) also showed significant enrichment. These terms suggest that, under this condition, the gene expression related to nucleic acid metabolism and phosphorus metabolism processes was significantly altered (Figure 2B). In the comparison between T6N and T6C, the extracellular region and serine-type endopeptidase inhibitor activity were significantly enriched again. Furthermore, there were other terms related to protein degradation and regulation, such as endopeptidase inhibitor activity (GO:0004866) and peptidase inhibitor activity (GO:0030414). This indicates that, under these conditions, the gene expression related to extracellular and protein degradation processes had significantly changed (Figure 2C). In the comparison between T24N and T24C, in addition to the extracellular region, terms related to carbohydrate metabolism, such as the aminoglycan catabolic process (GO:0006026) and chitin metabolic process (GO:0006030), also showed significant enrichment. This suggests that, under these conditions, the gene expression related to carbohydrate metabolism was significantly altered (Figure 2D). The commonly enriched term “extracellular region” appeared in all comparisons, emphasizing the widespread impact of needle pricking on gene expression related to the extracellular environment. However, under the same mechanical damage conditions, RH only showed enrichment of the “response to stress” (GO:0006950) term at 6 h (Figure 2A), while TN1 not only showed the enrichment of “response to stress”, but also “response to wounding” (GO:0009611) and “chitinase activity” (GO:0004568) at 6 h; at 24 h, TN1 showed the enrichment of “chitin metabolic process”, “chitin catabolic process”, “chitin binding” (GO:0008061), and “defense response to fungus” (GO:0050832) (Figure 2C,D). In summary, the insect-susceptible rice variety TN1 may need to mobilize more internal resources to cope with mechanical damage, thereby experiencing more complex stress responses, leading to more significant changes in resistance-related activities.

### 2.3. KEGG Pathway Enrichment Analysis of Differentially Expressed Genes in the Four Treatment Groups

**Figure 3:**
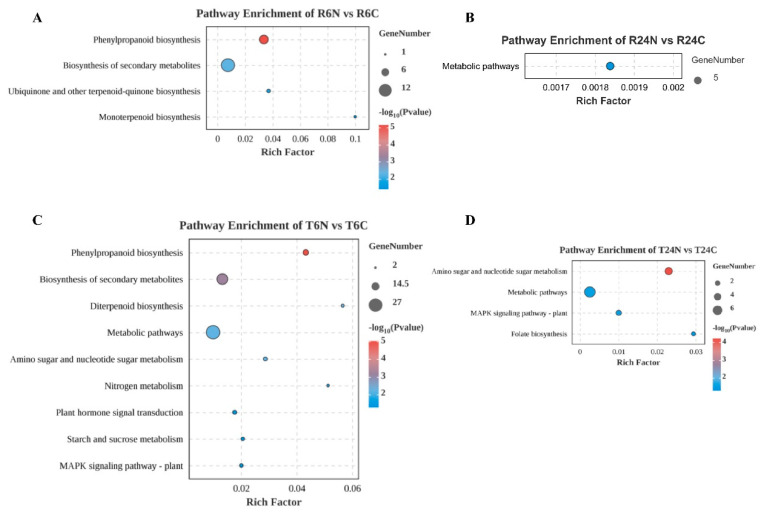
KEGG pathway enrichment analysis of differentially expressed genes in the four treatment groups. (**A**): KEGG pathway enrichment analysis of differentially expressed genes in R6N-R6C. (**B**): KEGG pathway enrichment analysis of differentially expressed genes in R24N-R24C. (**C**): KEGG pathway enrichment analysis of differentially expressed genes in T6N-T6C. (**D**): KEGG pathway enrichment analysis of differentially expressed genes in T24N-T24C.

In the comparison between R6N and R6C, the most significantly enriched KEGG pathway was Phenylpropanoid Biosynthesis (ko00940). This pathway is involved in the synthesis of phenylpropanoid compounds, which are a diverse group of organic compounds derived from phenylalanine. Phenylpropanoids play important roles in plant structure (e.g., lignin), defense (e.g., plant antibiotics), and signaling (e.g., flavonoids). The next was Biosynthesis of Secondary Metabolites (ko01110). This pathway covers the synthesis of a wide range of secondary metabolites, which do not directly participate in the normal growth, development, or reproduction of organisms. Secondary metabolites often play important roles in ecosystems, such as defense mechanisms and signaling. There was also enrichment of the Ubiquinone and Other Terpenoid–Quinone Biosynthesis (ko00130) and Monoterpenoid Biosynthesis (ko00902) pathways (Figure 3A). This suggests that the physical damage caused by needle pricking may have put RH in an active metabolic state, particularly related to defense mechanisms, energy metabolism, and secondary metabolite synthesis. As time increased, at 24 h, RH only had the Metabolic Pathways (ko01100) enriched (Figure 3B). RH may have an efficient defense strategy that can quickly process and repair the damage caused by needle pricking, reducing the long-term gene expression requirement. This indicates that the RH variety has a strong adaptive capacity when facing physical damage.

In contrast, for TN1, in addition to the large-scale enrichment of Phenylpropanoid Biosynthesis and Biosynthesis of Secondary Metabolites 6 h after needle pricking, a large number of DEGs were involved in Metabolic Pathways, and some DEGs were involved in defense- and injury-repair-related signaling pathways (Figure 3C). Moreover, 24 h after needle pricking, Amino Sugar and Nucleotide Sugar Metabolism (ko00520), Metabolic Pathways, MAPK Signaling Pathway—Plant (ko04016), and Folate Biosynthesis (ko00790) were enriched (Figure 3D). This indicates that 24 h after needle pricking, TN1 is still actively adjusting its metabolic activities and maintaining its defense and stress responses.

### 2.4. Expression Profiles of DEGs during Growth Stages and in Response to Hormones

In transgenic breeding, inducible promoters can be used to control the expression of insect resistance genes when under pest attack, thereby improving crop insect resistance. For this purpose, we should choose genes with low basal expression in order to minimize unnecessary gene expression interference during normal growth stages. Additionally, these genes should show significant inducible expression upon treatment with resistance-related hormones (such as ABA, IAA, and JA).

**Figure 4:**
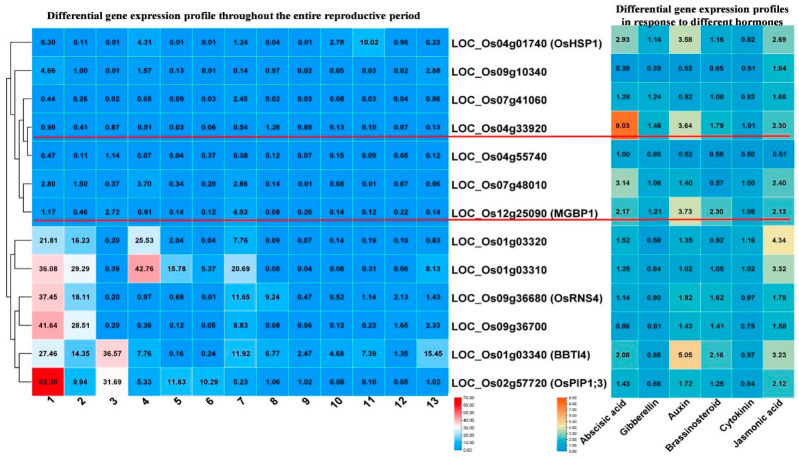
Expression profiles of 13 DEGs during growth stages and in response to hormones. The heatmap on the left shows the expression patterns during growth stages, where 1: leaf and root at three-leaf stage; 2: shoot, seedling with 2 tillers; 3: root, seedling with 2 tillers; 4: leaf, young panicle shorter than 1 mm.; 5: flag leaf, 5 days before heading; 6: flag leaf, 14 days after heading; 7: sheath, panicles shorter than 1 mm; 8: stem, 5 days before heading; 9: stem, heading stage; 10: panicles shorter than 1 mm.; 11: panicle (10 to 30 mm); 12: panicle (40 to 50mm); 13: panicle, heading stage. The heatmap on the right shows the hormone response expression patterns, where Abscisic acid: ABA; Gibberellin: gibberellic acid, GA3; Auxin: indole-3-acetic acid, IAA; Brassinosteroid: brassinolide, BL; Cytokinin: trans-zeatin, tZ; Jasmonic acid, JA.

Based on the transcriptome data from the four treatment groups, combined with gene function prediction, we screened out 13 DEGs with an induction fold change greater than four-fold. Further heatmap clustering analysis of the expression patterns of these 13 genes showed that seven DEGs had relatively low basal expression across various rice tissues from the seedling stage to the heading stage, while the remaining six DEGs had relatively high basal expression in some tissues at certain stages (Figure 4).

When analyzing the expression profiles of these 13 DEGs under exogenous hormone treatments, we found that these genes responded to the hormone treatments. Our research goal is to identify genes that have low basal expression in various tissues during normal growth stages but can be induced by resistance-related hormones. Therefore, among the seven DEGs with low basal expression, we selected the promoters of *LOC_Os04g33920* (P1) and *LOC_Os12g25090* (*MGBP1*) (P2) for further experimental validation (Figure 4).

### 2.5. GUS Analysis of Candidate Promoters

We constructed expression vectors containing the GUS reporter gene to validate the functions of the two candidate promoters (P1 and P2), using the 35S promoter as a positive control. Through RT-PCR and GUS staining experiments, we systematically evaluated the expression characteristics of these promoters in transgenic plants.

**Figure 5:**
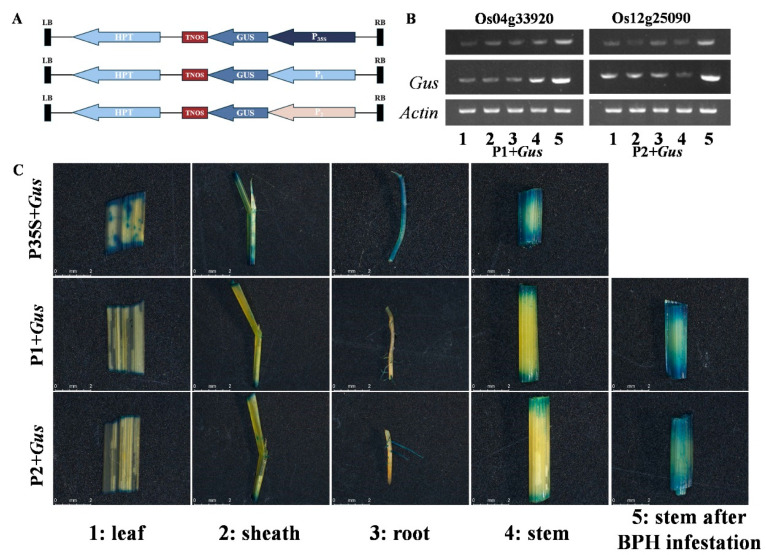
GUS analysis of candidate promoters. (**A**): Construction of GUS reporter gene expression vectors. (**B**): RT-PCR results, where the numbers represent different tissues and treatments: 1: leaf; 2: sheath; 3: root; 4: stem; 5: stem 24 h after BPH infestation. (**C**): GUS staining results driven by different promoters.

First, we constructed three vectors, P35S:GUS, P1:GUS, and P2:GUS, all containing the Hygromycin Phosphotransferase (HPT) gene as a selection marker. The *GUS* gene in each vector was driven by the 35S promoter, P1, and P2, respectively (Figure 5A, Supplementary S1 and S2). Through genetic transformation, we successfully obtained transgenic plants carrying different vectors and confirmed the transgenic plants through resistance screening and PCR identification.

Next, we performed RT-PCR experiments to detect the expression level of the *GUS* gene in the transgenic plants with different vectors. The results showed that *GUS* gene expression could be detected in all transgenic plants. The RT-PCR results indicated that under normal conditions, the endogenous *LOC_Os04g33920* and *LOC_Os12g25090* (*MGBP1*) genes, as well as the *GUS* genes driven by the P1 and P2 promoters, had relatively low expression levels. However, after brown planthopper (BPH) infestation, their expression levels were significantly upregulated, showing an inducible characteristic (Figure 5B). Furthermore, we further verified the *GUS* gene expression through GUS staining experiments. The results showed that the *GUS* gene driven by the 35S promoter had widespread and strong expression in various plant tissues. In contrast, the GUS genes driven by the P1 and P2 promoters had low-level expression in different tissues, and the staining intensity in the stem only significantly increased after BPH infestation, indicating their BPH-inducible expression characteristics (Figure 5C). These results suggest that these promoters have the potential for application in insect-resistant breeding, as they exhibit low basal expression and strong inducibility.

### 2.6. Expression of OsLecRK1* Gene Driven by Promoters and Resistance Evaluation

**Figure 6:**
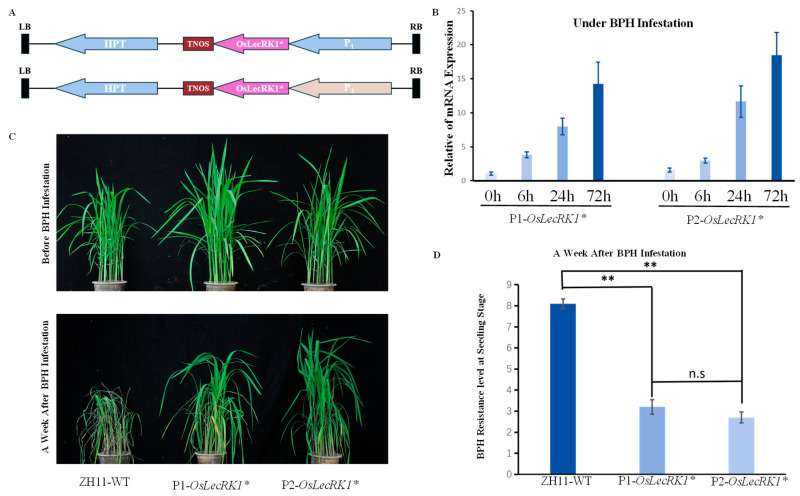
Construction of the expression vector for OsLecRK1* gene and resistance evaluation. (**A**): Construction of the expression vector for OsLecRK1* gene. (**B**): Expression of OsLecRK1* gene after BPH infestation. (**C**): Effect on plants a week after BPH infestation. (**D**): BPH resistance level of P1-OsLecRK1* and P1-OsLecRK1* at seeding stage. The numbers on the *Y*-axis represent the resistance level of brown planthoppers, 0: immunity to BPH; 1: high resistance; 3: resistance; 5: moderate resistance; 7: susceptible; 9: highly susceptible, ** means very significant difference (*p* < 0.05), n.s means no statistical difference.

The major resistance gene against brown planthopper (BPH) in rice, *Bph3*, is composed of a gene cluster of three *LecRK* genes, namely, *OsLecRK1*, *OsLecRK2*, and *OsLecRK3*. Among them, *OsLecRK1* has the greatest anti-BPH effect. Therefore, we constructed two expression vectors containing the OsLecRK1* gene (*: codon-optimized), driven by the candidate promoters P1 and P2, and obtained the corresponding transgenic plants through genetic transformation (Figure 6A, Supplementary S2 and S3). To evaluate the effects of these promoters in driving the expression of and enhancing plant resistance, we performed a qRT-PCR expression analysis and resistance evaluation experiments.

The qRT-PCR results showed that the expression level of the OsLecRK1* gene was significantly increased under BPH infestation conditions, and it exhibited high expression levels when subjected to BPH infestation (Figure 6B). This indicates that the two candidate promoters can effectively induce the expression of the OsLecRK1* gene. The resistance evaluation further confirmed the function of the OsLecRK1* gene. Under BPH infestation, the wild-type ZH11 showed obvious growth inhibition and leaf yellowing, while the transgenic plants exhibited significant resistance and maintained good growth (Figure 6C). The statistical results of resistance indicate that OsLecRK1* driven by both P1 and P2 promoters can reach the resistance level to brown planthoppers (Figure 6D, Supplementary S4). These results suggest that the P1 and P2 promoters have good potential in driving the expression of the OsLecRK1* gene and enhancing the resistance of rice to BPH.

## 3. Discussion

In the field of plant biotechnology, genetic engineering is the main tool used for crop improvement and gene function research, and promoters are the primary tools for crop improvement. Commonly used constitutive promoters include the CaMV 35S, the *actin* gene promoter Act, and the ubiquitin gene promoter Ubi [28,29,30]. However, due to the impact of constitutive promoters on cellular function or high energy consumption, the expression of exogenous genes under the transcriptional regulation of constitutive promoters often leads to unexpected phenotypic changes. For example, the constitutive expression of stress-tolerant transgenes, such as CaMV35S:DREB1A in *Arabidopsis* [31], CaMV35S:CBF1 in transgenic tomato lines [32], and CaMV35S:ADC in transgenic rice [33], can result in growth retardation, developmental abnormalities, and reduced seed production. Similarly, the overexpression of *OsNAC6* driven by the maize ubiquitin promoter in transgenic rice plants led to severe developmental defects [34]. In contrast, the expression level of the rice *BPH14* gene driven by its own promoter was much lower than that driven by the Ubi promoter, but both conferred the same level of insect resistance [35]. Moreover, in multi-gene transformation events, the use of the same strong constitutive promoter to drive the expression of multiple target genes can increase the occurrence of homology-dependent gene silencing [36,37,38]. Constitutive expression can also impose a physiological burden on plants. Therefore, the combined use of constitutive, inducible, and tissue-specific promoters can effectively reduce these problems.

Currently, as the research on promoters and cis-regulatory elements deepens, an increasing number of inducible and tissue-specific promoters and cis-regulatory elements are being discovered. A 193 bp region in the promoter of the wheat *TaNRX1-D* gene has significant responses to osmotic stress and ABA treatment, and a 36 bp region (−193 to −157 bp) is considered to be the key sequence for the *TaNRX1-D* gene to respond to PEG6000 or ABA treatment [39]. Bioinformatics and transgenic experiments on the promoters of *OsAOS1* and *OsHPL2* genes have identified multiple promoter regions responsive to brown planthopper and rice stem borer, and the combination of different regions has found the promoter sequence with the best induction effect on downstream gene expression [40,41]. However, these types of promoters have not been widely adopted in crop genetic engineering breeding research.

The study of insect-feeding-inducible promoters lags far behind that of abiotic stress and microbial-stress-inducible promoters. On the one hand, it is because the most convenient control effect of insect damage is still chemical pesticides, and there are mature constitutive promoters that can be used. On the other hand, the relevant research needs to have enough test insects to use. Due to the randomness and uncertainty of insect feeding, the experiment needs more repeated processing to ensure the accuracy of the results. Compared with abiotic stress and microbial stress, the research of insect-feeding-inducible promoters is slower and more difficult. It is hoped that more people will pay attention to insect-feeding-inducible promoters in future research and provide more convenient scientific research tools for plant transgenic insect resistance research and breeding. Endogenous insect-feeding-inducible promoters and cis-regulatory elements have many defects in both expression level and spatio-temporal regulation. Therefore, in future promoter research, it is necessary to artificially improve the promoters related to insect feeding induction; assist with enhancers and the tissue-specific expression of cis-regulatory elements or promoter, core promoter, and other promoter components; and artificially design and synthesize mature promoters that can meet the application standards.

This study conducted transcriptome analysis at 6 h and 24 h after wounding treatment on the resistant rice variety RH and the susceptible variety TN1. We identified 60 differentially expressed genes that were induced by wounding in both RH and TN1. Based on functional prediction, we further selected 13 of these genes for expression profiling analysis. Aiming to screen for inducible promoters, we identified two genes, *LOC_Os04g33920* and *LOC_Os12g25090* (*MGBP1*), that showed low basal expression but were significantly induced by defense-related hormones such as ABA, IAA, and JA. We successfully isolated two rice wound-inducible promoters, P1 (*LOC_Os04g33920*) and P2 (*LOC_Os12g25090*), from these two genes. The products of *LOC_Os04g33920* and *LOC_Os12g25090* (*MGBP1*) are both protease inhibitors. Numerous studies have shown that plant protease inhibitors can inhibit the digestive proteases of various insects, disrupting their normal growth and development, thereby exhibiting significant insecticidal activity. The efficacy of different protease inhibitors varies depending on the major protease types in the target insects (*Lepidoptera*, *Coleoptera*, *Diptera*, *Hemiptera*). Serine protease inhibitors can regulate the activity of serine proteases, affecting the growth and development of *Dipteran* insects [42] as well as the larval weight, development time, adult emergence, fecundity, and survival rate of *Lepidopteran* insects [43]. Cysteine protease inhibitors, on the other hand, can disrupt the normal growth and development of *Coleopteran* insects by hydrolyzing their cysteine proteases [44]. As the major digestive proteases in most Lepidopteran insects are serine proteases, while those in most *Coleopteran* insects are cysteine proteases, cysteine protease inhibitors generally exhibit better insecticidal effects against *Coleopteran* pests than serine protease inhibitors. Protease inhibitors from non-host plants have also shown significant insecticidal activity against phytophagous insects [45,46]. Both serine and cysteine protease inhibitors can reduce the body weight, prolong the development time, and decrease the population density of aphids [47]. Therefore, protease inhibitors also have potential for the control of piercing–sucking insects such as the brown planthopper. We predicted the cis-acting elements of these two promoter sequences (Supplementary S5) and found that there were multiple cis-regulatory elements in these two promoters that could be induced by biotic or abiotic stresses.

**Figure 7:**
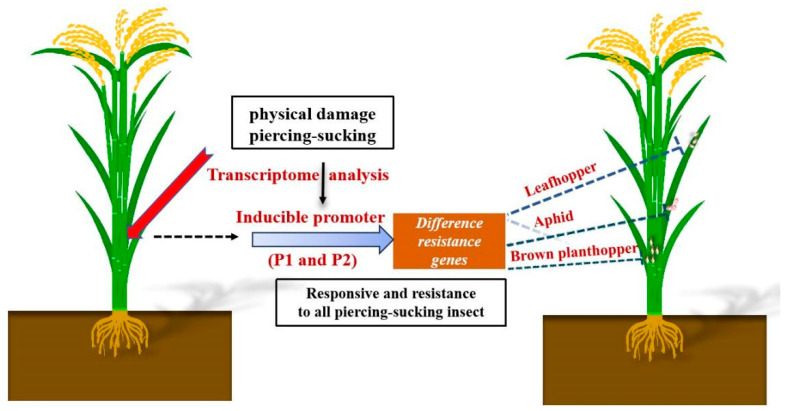
Schematic diagram of rice defense against piercing–sucking insects induced by acupuncture physical damage.

In theory, these two promoters should respond to the feeding of all rice-feeding piercing–sucking insect pests. We also verified this in our subsequent validation experiments using the GUS reporter gene and an insect resistance gene driven by these promoters. Our results show that, under normal conditions, the expression levels of the *GUS* gene and OsLecRK1* driven by the P1 and P2 promoters are relatively low, but they are significantly upregulated upon infestation by the brown planthopper, demonstrating their inducible characteristics. This indicates that these two genes can be useful for the genetic engineering of rice resistance, especially against piercing–sucking insect pests. If suitable genes are available, these promoters can not only be explored in the brown planthopper, but also in other piercing–sucking insect pests, such as leafhoppers (Figure 7).

## 4. Materials and Methods

### 4.1. Experimental Materials

Rice: The BPH (brown planthopper)-resistant variety Rathu Heenati (RH) and the susceptible variety Taichung Native 1 (TN1) used in this study were sourced from the resources preserved by our research group (Food Crops Institute, Hubei Academy of Agricultural Sciences). Additionally, the rice variety Zhonghua 11 (ZH11), used for genetic transformation, was also collected from our laboratory’s rice varieties [48].

Brown planthopper: The brown planthoppers used in this study were collected from experimental fields in Wuhan, China. They were reared indoors for more than 10 generations using the susceptible rice variety TN1 seedlings. The rearing conditions were 27 ± 2 °C, relative humidity 65 ± 5%, and a photoperiod of 16 h light and 8 h dark [26].

### 4.2. Preparation and Treatment of Rice Samples

The seeds of the TN1 and RH rice varieties were soaked at 37 °C in an incubator for 2 days, followed by a 2-day germination period. Subsequently, the seeds were sown into small red pots, with 15 seeds per pot. After 2 weeks of growth, the best 10 seedlings were retained in each pot. During the seedling stage, sterile needles were used to induce pricking, representing pure mechanical damage. Each plant was pricked 15 times at the bottom of the seedling with a needle and then grew normally. Samples were collected at 6 h and 24 h after the pricking treatment, resulting in a total of 8 sample groups and 24 transcriptomes. The 8 sample groups were designated as R6C, R24C, R6N, R24N, T6C, T24C, T6N, and T24N. Here, R denotes the resistant rice variety RH, T denotes the susceptible rice variety TN1, 6 indicates the 6 h time point, 24 indicates the 24 h time point, C represents the untreated control samples, and N represents the samples subjected to simulated mechanical damage. Each processing and sampling time point is repeated three times. The total RNA from all 24 rice samples was extracted using TRIzol reagent (Invitrogen, Waltham, MA, USA), and whole-genome expression analysis was conducted by CapitalBio Corporation, Beijing, China.

### 4.3. Venn Diagram, Expression Pattern Clustering, KEGG, and GO Analysis of Transcriptome Data

Transcriptome data analysis and differential expression gene (DEG) identification were performed using TBtools v2.096 software. A Venn diagram was created using the Venny 2.1 tool (https://bioinfogp.cnb.csic.es/tools/venny/, accessed on 1 July 2024), and the DEG expression patterns for systematic cluster analysis were developed using the website https://www.tm4.org/mev/ (accessed on 1 July 2024). All DEGs were used for Gene Ontology (GO) and Kyoto Encyclopedia of Genes and Genomes (KEGG) enrichment analysis. GO analysis using the GO database (https://www.geneontology.org, accessed on 1 July 2024) demonstrated the biological significance of the identified DEGs. TBtools software was used to measure the statistical enrichment of DEG in the KEGG pathway (https://www.kegg.jp/kegg/kegg2.html, accessed on 1 July 2024), and padj < 0.05 was used as the enrichment criterion. Finally, the results were drawn by using the online tool at https://www.omicshare.com/tools/ (accessed on 1 July 2024).

### 4.4. Expression Profiling of Differential Genes across Developmental Stages and Hormone Responses

The whole growth period expression data of the DEGs were obtained from CREP (Collection of Rice Expression Profiles) (https://crep.ncpgr.cn/, accessed on 1 July 2024), and the hormone response data were obtained from RiceXPro (https://ricexpro.dna.affrc.go.jp/, accessed on 1 July 2024). Then, TBtools was used to draw the heatmap.

### 4.5. Construction of Promoter Vectors for Candidate Genes

Based on the reference genome information for Japonica rice provided by the Rice Genome Annotation Project (https://rice.plantbiology.msu.edu/, accessed on 1 July 2024), about a 2000 bp upstream sequence of the genes *LOC_Os04g33920* and *LOC_Os12g25090* was searched using NCBI (https://www.ncbi.nlm.nih.gov/, accessed on 1 July 2024) as the promoter sequence, and Primer Premier 6.0 was used for primer design. Then, the promoter sequences for the genes *LOC_Os04g33920* (P1) and *LOC_Os12g25090* (P2) were cloned. The lengths of these promoter sequences are 1626 bp and 1959 bp, respectively (Supplementary S1). Using the genome of rice variety ZH11 as a template, the promoters were amplified and subsequently linked to the GUS gene and the codon-optimized (*) brown planthopper resistance gene OsLecRK1* (Supplementary S3) [49]. The primer and the process of construction vectors is shown in Supplementary Material S2.

### 4.6. Agrobacterium-Mediated Genetic Transformation of Rice

The successfully constructed promoter vectors and brown planthopper resistance vectors were introduced into *Agrobacterium* strain EHA105 using the rice variety ZH11 for callus tissue culture and genetic transformation. The HPT gene was used as a marker, and the T_0_ transgenic plants were obtained via PCR-positive detection for *hygromycin* B screening. The T_1_ generation transgenic homozygous-positive plants were screened using the germination test on the medium containing screening reagent. Finally, the T_2_ single-copy homozygous *Gus* or OsLecRK1* promoter family was used for the next experiment [50,51].

### 4.7. GUS Histochemical Staining of Transgenic Plants with Promoter Constructs

Rice tissues, including the leaves, leaf sheaths, roots, and stems from both brown planthopper-fed and untreated plants, were collected and incubated in staining buffer at 37 °C overnight. GUS staining buffer was prepared following the method described by Jefferson et al. (1987). The rice tissues were then decolorized in 75% and 95% ethanol at 37 °C, with each decolorization step lasting 3 h and repeated 3–5 times. The decolorized tissues were stored in 75% ethanol and examined under a Leica MZ FLIII dissecting microscope (Leica Microsystems, Wetzlar, Germany). Images were captured using a Nikon E5400 camera (Chiyoda, Japan).

### 4.8. RNA Extraction, Reverse Transcription, and Expression Analysis in Rice Tissues

The rice tissue samples were immediately frozen in liquid nitrogen upon collection. The total RNA was extracted using TransZol reagent (TransGen Biotech, Beijing, China). For reverse transcription, 2–3 µg of total RNA [RNA integrity number (RIN) > 7] was treated with DNase I (Invitrogen, Carlsbad, CA, USA) to remove any DNA contamination. The first-strand cDNA was synthesized using the M-MLV reverse-transcriptase kit (Invitrogen, Carlsbad, CA, USA).

The rice *actin* gene (*Actin*) was used as an internal reference gene. PCR amplification of the cDNA was performed using KOD DNA polymerase, followed by RT-PCR gel electrophoresis for detection.

Quantitative real-time PCR (qRT-PCR) was conducted using the FastStart Universal SYBR Green Master (Roche, Basel, Switzerland) kit on the ABI 7500 system (Applied Biosystems, Foster City, CA, USA). The relative expression levels of the target genes were calculated using the 2^−ΔΔCt^ method [52], with the rice *actin* gene (*Actin*) as the internal reference.

## 5. Conclusions

Through the comparative transcriptomic analysis of two rice varieties under needle-wounding stress, this study found that the insect-susceptible variety TN1 exhibited a more pronounced stress response, requiring the mobilization of more internal resources to cope with the damage. Further screening identified two promoters, P1 and P2, with inducible expression characteristics, which could effectively drive the expression of the insect resistance gene OsLecRK1* and enhance the resistance of transgenic plants to the brown planthopper. These findings provide promoters for the development of insect-resistant transgenic crops.

## Data Availability

All eight samples (24 transcriptomes) were submitted to the NCBI website (http://www.ncbi.nlm.nih.gov/geo/, accessed on 1 July 2024; accession number: GSE74106).

## Supplementary Materials

The following supporting information can be downloaded at: https://www.mdpi.com/article/10.3390/ijms251910564/s1.
