# Supplementary material for: Identification, Cloning, and Characterization of Two Acupuncture-Injury-Inducing Promoters in Rice
Source: Int J Mol Sci. 2024 Sep 30;25(19):10564. doi: 10.3390/ijms251910564 (PMC11476359; doi:10.3390/ijms251910564)
Supplement: Supplementary file 1 [file ijms-25-10564-s001.zip › Supplementary S1 Sequences of P1 and P2.pdf]

>P1: Promoter of LOC_Os04g33920

ctacacgagcagaccactggctgcttcccgtcgaccaggccgctgccgctcccttcctctgtccccaccgccaccgccgcc
actccccaccgccgctccggccggggtgaagagaaacgctgggggagagagggaaggagggaggaggaagaag
ggaagccgggaaggagctagagggaggatgacatgtgggtctcacatatcaatggatcccacaatataatttttgtgtg
agtgacatgtaggtcctaccttttttttatttttattctaatgccacataagcgccacgtagaacaaagacttggtcaataccgc
cacgtaggcgccacgtcagctaaaatcaccgagggatataatttgcaccggttttgatagttggaggagtcaatttacctg
gttttgtggttaaaggatatgaatcatactcggagctatagttgagggagtcaaagtatatttttccatcccaattgtcccacc
cacctgatactaccatcacgcgacgcgacgccccacggccggcggcgcgaatcgcctaccgcccgctgcacagg
ggcactagacacacatgggctgatgggcacatgacttagcccagcattctcgcttacatatcttatgttgtttttttattaaga
cgtggattttaccaaggattttattagtatgttttctaaaccgctaaacaatatgtttttcaaaaaattatataaaaataattttaa
aatatcaaataaatttatttttaagtttgtaacaattaaaacttaattaatcatgtgactttttcattttacgtatgctaacttaatcta
tatccaactctattttaattatatgttaatggctttttttattttaggtagactatcttaatcttcgtccaactctattttttaatttttaag
tttgtaataatactgcccaacacggccaggatgaagcgtcgatctgaatttctttttcaattttgagggcactctccttctaga
gacactcccttatgaacggagcatcgaatttgggattgtggcaaatggcaatgcaccgttgctgtgcacaggtgccggcc
gggcgcgcttacacggctacacctaacacgcctacactctttttttctcatcagtcatcagaatacttacacgtttacatttgtgc
ttttgacaatcctagtaggttataattagtactaagtgcttagccgatgaacgaaatgatgaccaacaccaccgacacacca
ccaaaactcttgtatggatgtttttctgttgtttgcatatcatttaaatagttacaaataaattaataaaaactagaagatatatta
atatgtgatatatcacttcataaacacgtaacttaacatttaatttctacttgtaagtttaatttattttttgttgtcatatacagaag
ttaaattttaagttgtatgtttgtagagtgatatatcacatgttaatatatttttttaaaagctttttataactatttgagtagtatgca
agtaaggagtggaagtaaggagtggacgttcaaaactagtactcgctaaactagtcgagatctctttttctctaccgctgc
gcaacaacatttgattgaaccatcttacgatgct

>P2：Promoter of LOC_Os12g25090（MGBP1）

taccaccgtgtggcactaggctggcaggcaagggggggcggtgggccggcccatccggccctcagcaaaaaggcagg
ggaagccgtcggggggttagggttaaccctaaccgcccccacgatttcgctgctgccgccgttttcgccgctcaaaccgc
caccctttttcgccgcacaccgccgccatttcgctgctaccgatgccgccgttgggcgccaccgacgccgccgaggggac
gccgagggggcgctgcccttcctccgccgccgtggggcgccaacgacgccaccgaggggggcgccgcccttcctctg
ccgccgccgcaccatctgccgcccccatctccccgttacggcgccgatgcatgatgcgccgccgccgctgcctccatatcc
gccgccgtccaccaccgcttgcatccacaatccgctacccaccgttgtcgccatccaccatccactgccgccacggccgcc
cctccgtccgccggcggtttgccggccggattaggccgacggctcccgatcctcatcggaatcggaggccggccgagc
ccggacgccattccccggcaccccgttagggagtgtacggctaggagagccatggggaatggcgatgaaggtgtggc
ggaggccgggacggcaaacggcggcttcacctgcatgacgacgagacgaggacggattgaagaagggcggctccc
ccgtgcgcgcagccagggtaccggttctgccggaccccagtagcgcgggagtgggggaacggcgtcggcgtcggct
tggtgtcggtggaggtggtgaaggggcggtcagtggtggcggcagcgactccatctccagggaaaggtcacatggct
cgccgggcagtgtcaccactgccgtccatccgtggtaggcgcagtactgcaccccctgcacacagatacggcgaggca
cggcggcaaccggagacggaggcggaggaggagcagccggagtagtggccaccacgatgcgtcggcgagggtg

gtggcgacggccaccagccctagcccgacgttcatggtgttagtggcgaccgtggtggtggcggcgactcctctccgat
cgacggagaagatggtgaaggcgacgggcgcgctgattgcgagggaggccgagaacggcatccataatccaatga
aaggtaggcatcgtaccttctctgttgaatctcctgttcgcttttctgttagatcaaaagcgatgtttcttccttctttcacttcgc
gatctccttcgcgagagcgaaagtgctgataacgtgttgaatgtggatagaattgattgagcgagagagagaattgaac
acaaacagagcgaatcgatagatttctcattgatcaacagtacatttatacaatgtgaagcggtgtcaactgcaggggac
gcgatatttaggaacatcgcgtcggttgtcgagagggacgctaaagggagagagaggagcgatctcctcttctctaccg
gttagtataggaaccgttgttcctatttccttgggtggatgagatcacccgtagtttcttaacaaaaggagcggaattgttgt
gtgagaaaccaaactggtttgttgttcgtgtggacactggacagagctgttgactgatcgatggtggtgcggtggtgcca
tttcaaacttggcaaaagtaggtgacacggtagttgaatgttttgtacgtagtggccggccgctggggccacgagcgtgc
cacgccatcatcaaacacgtttctatctttttctttttggaggtttcgttttctcttggtcaacattaatccgctcgatcgccacgtg
atcctgttgtgtcttaattactcgacgctttgatcaaacagcttgaccagcaacgtcgtgtggcatatgcatgcaggcacgta
cacagtcagatccaccttggcaagctataatcctgatccaaacctgcctctggaactc
