# Supplementary material for: Identification, Cloning, and Characterization of Two Acupuncture-Injury-Inducing Promoters in Rice
Source: Int J Mol Sci. 2024 Sep 30;25(19):10564. doi: 10.3390/ijms251910564 (PMC11476359; doi:10.3390/ijms251910564)
Supplement: Supplementary file 1 [file ijms-25-10564-s001.zip › Supplementary S2 All primers used in this study and the process of construction vectors.pdf]

Primers used in this study		
Purpose	Primer(Gene Name)	Primer sequence (5' -3')
promoter cloning and vectors construction	*P1*	F: ctgcagCTACACGAGCAGACCACT
		R: aagtccAGCATCGTAAGATGGTTCAA
	P2	F: ctgcagTACCACCGTGTGGCACTA
		R: aagtccGAGTTCCAGAGGCAGGTT
	*OsLecRK1**	F: aagtccATGGTGGCCCTCCTGCTCTT
		R: ggtaccCTACGGGAGAGAGCTGAT
	TNOS	F: aataccGATCGTTCAAACATTTGGC
		R: gaattcGATCTAGTAACATAGATG
qRT-PCR	*q-p1*	F: GACAAGATGGCGCTGAAG
		R: TCATGGCAGAGTGTAATCTC
	q-p2	F: ACCTGCGTCAATGTAATGTTCTG
		R: TCATGGCAGAGTGTAATCTC
	Gus	F: ACCTGCGTCAATGTAATGTTCTG
		R: TTCGGTGATGATAATCGGCTGAT
	Actin	F: TGGCATCTCTCAGCACATTCC
		R: TGCACAATGGATGGGCCAGA
	*q-OsLecRK1**	F: TACCCGAAGAAGGCAAATGC
		R: GAAGCAGTCGATCACGCACA
positive detection	*HPT*	F: ACACTACATGGCGTGATTTCAT
		R: TCCACTATCGGCGAGTACTTCT

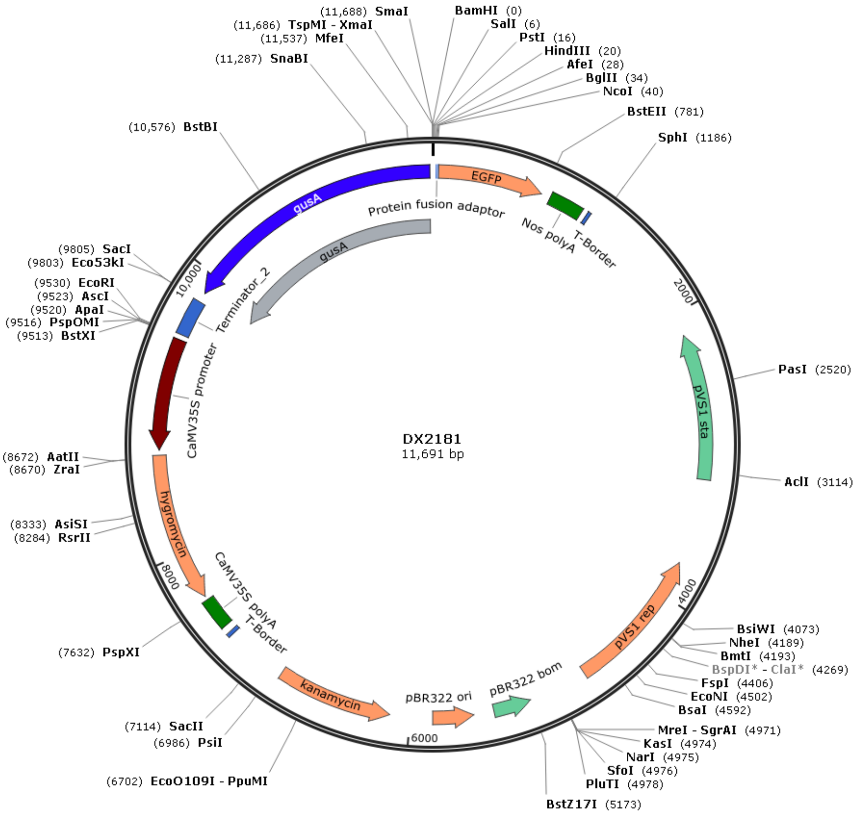

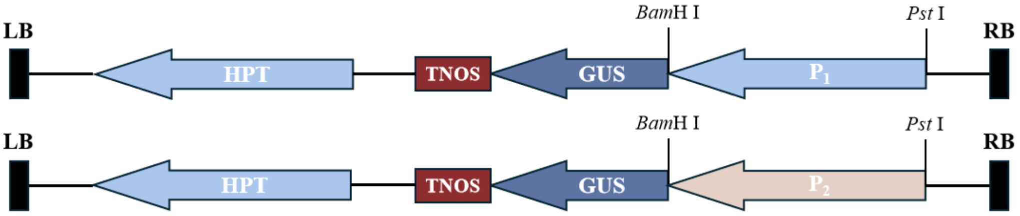

P1 and P2 were amplified by P1 and P2 primers, respectively, and then linked to vector DX2181 using *Pst* 1 and *BamH* 1 double enzyme cleavage. Obtained vectors with P1 and P2 promoters respectively driving the expression of the reporter gene *Gus* for subsequent research.

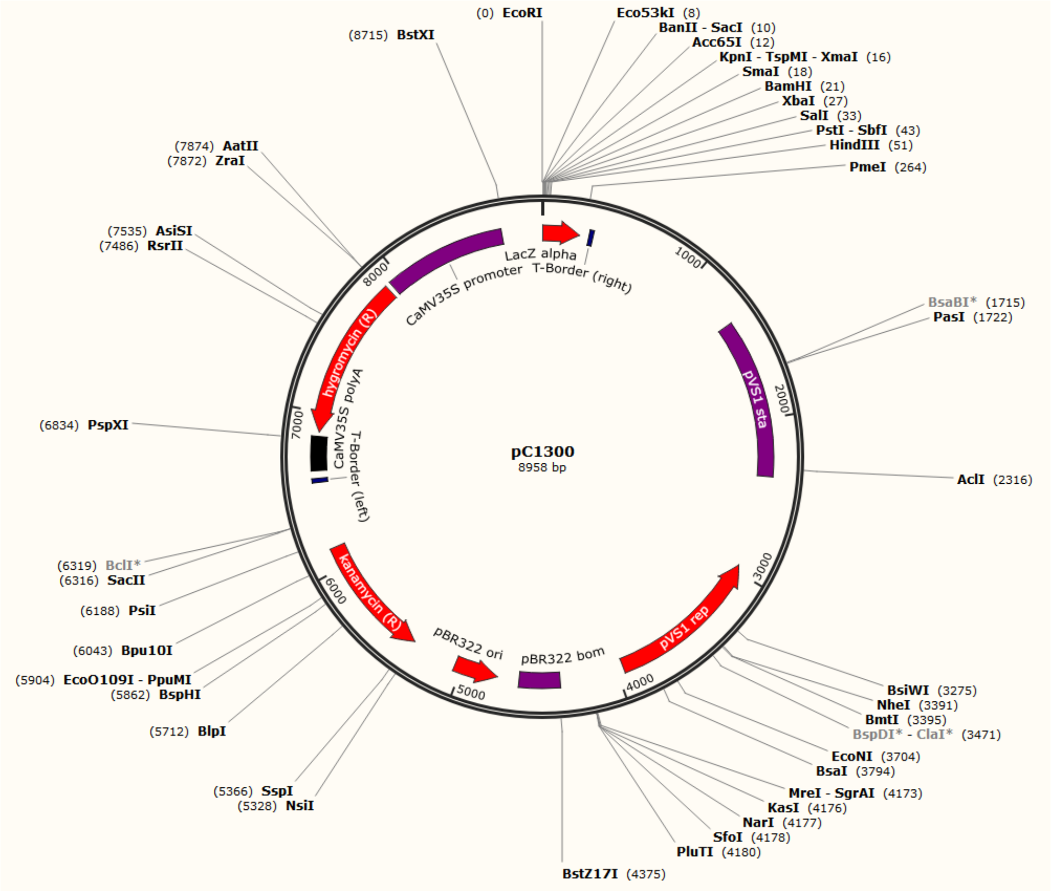

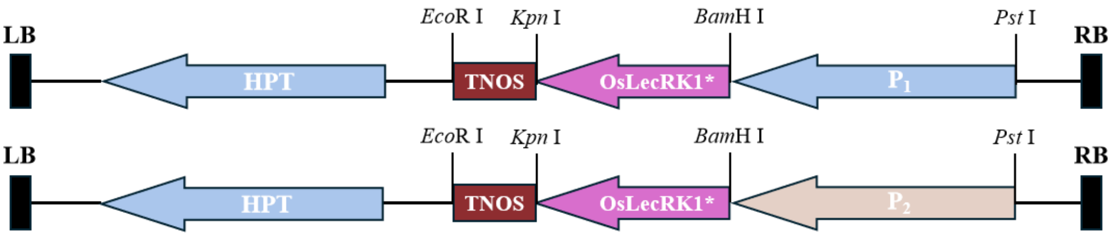

Firstly, P1 and P2 were amplified by P1 and P2 primers, respectively, and then linked to vector PC1300 using *Pst* 1 and *BamH* 1 double enzyme cleavage.

Secondly, *OsLecRK1** were amplified by *OsLecRK1** primers, and then linked to vector PC1300 using *BamH* 1 and *Kpn* 1 double enzyme cleavage.

Last, Nos terminator was amplified by *OsLecRK1** primers, and then linked to vector PC1300 using *Kpn* 1 and *EcoR* 1 double enzyme cleavage.

Obtained vectors with P1 and P2 promoters respectively driving the expression of the BPH-resistace gene *OsLecRK1** for subsequent research.
