# Supplementary material for: Identification, Cloning, and Characterization of Two Acupuncture-Injury-Inducing Promoters in Rice
Source: Int J Mol Sci. 2024 Sep 30;25(19):10564. doi: 10.3390/ijms251910564 (PMC11476359; doi:10.3390/ijms251910564)
Supplement: Supplementary file 1 [file ijms-25-10564-s001.zip › Supplementary S3 BPH-resistance gene OsLecRK1 sequence optimized for codons.pdf]

> *OsLecRK1**

ATGGTGGCCCTCCTGCTCTTCCCAATGCTGCTCCAGCTGCTCTCCCCTACGT

GCGCGCAGACCCAGAAGAATATCACACTGGGCAGCACGCTCGCGCCACAG

TCTCCGGCGAGCAGCTGGCTCTCACCTTCCGGCGACTTCGCCTTCGGCTTC

AGGCCAGTGGAGGGCAACACGAGCTTCTACCTGATCGCCGTCTGGTTCAAT

AAGATCTCTGACAAGACGGTGGTCTGGTACGCGAAGAACACCGACCAGGA

CCCGAGCATCGTGGAGGTCCCATCCGATAGCTTCCTCCAGCTGACCAACGA

TGGAGCCCTCTCCCTGAAGGATAGGAGCGGCCAGGAGGGCTGGAATCCAC

AGGTGACATCTGTCGCCTACGCGTCAATGCGCGACACGGGCAACTTCGTGC

TGCTCGGCGCCGATGGCACCACAAAGTGGCAGACATTCGACATGCCTTCCG

ATACCATCCTCCCTACACAGGTCATCCCGTGCAACAAGACCAGGAATAAGA

GCCTGAGGGCCAGGCTCGACATCAATGATTACTCTTCAGGCAGGTTCCTGC

TCGACGTGCAGACCGATGGCAACCTCGCGCTGTACCTCGTGGCCGTCCCAT

CTGGCTCAAAGTACCAGCAGTACTGGTCAACGGATACGACCGGCAATGGCT

CGGAGCTCGTGTTCTCCGAGACCGGCAAGGTCTACTTCGCCCTGACAGAC

GGCACGCAGATCAACATCTCCTCTGGAGCCGGAATTGGCTCCATGGCAGAT

TACTTCCATAGGGCCACCCTCGACCCTGATGGAGTCTTCAGGCAGTATGTGT

ACCCGAAGAAGGCAAATGCCGGCATCCTGGGCGGCGAGACATGGACCGCC

GTGAGCATGCAGCCGCAGAACATCTGCCATGCGATTGTGTCAGATGTGGGC

TCGGGAGTCTGCGGCTTCAACTCATACTGCACGTTCGATGGCACCCGGAAT

CAGATCGCCTCGTGCCAGTGCCCGCCATGGTACAAGTTCTTCGACGAGCAG

AAGAAGTACAAGGGCTGCAAGCAGGATTTCCAGCCGCACTCGTGCGACCT

CGATGAGGCAACTGCCCTGGCACAGTTCGAGCTCCGCCCTATCTACGGCGT

GGACTGGCCGCTCTCCGATTACGAGAAGTACGAGCCAATCGGCCAGGACG

ATTGCGGCAGGCTGTGCGTGATCGACTGCTTCTGCGCGATGGCCGTCTACA

ATCAATCGACCTCCACATGCTGGAAGAAGAAGCTCCCTCTCTCCAACGGCA

ATATGGCCGATTACGTGCAGCGGACCGTCCTGCTCAAGGTGCCGAGCTCTA

ACTCATCGCAGAGCATGATCTCTACATCCAGCAACAAGTGGAAGAGGAATC

GGAAGCATTGGGTGCTCGGCTCTTCACTGATCCTCGGCACATCGATCCTGG

TCAATTTCGCGCTCATCTCCATCTTCCTGTTCGGCACCTACTGCCGCATCGC
CACAAAGAAGAACATCCCACTCTCACAGGCGTCGTCCAAGTCGCAGCTGC
CTCTCAAGACGTTCACCTACAAGGAGCTCGAGAAGGCGACTGCGGGATTC
CATGAGATCCTGGGAGCCGGAGCATCTGGCGTGGTCTACAAGGGCCAGCT
GGAGGACGAGCTCAAGACAAACATCGCCGTGAAGAAGATCGATAAGCTCC
AGCCGGAGACGGAGAAGGAGTTCATGGTCGAGGTCGAGACAATCGGCCA
GACGTTCCATAAGAACCTCGTGCGGCTGCTCGGCTTCTGCAATGAGGGAGC
CGAGAGGCTGCTGGTCTACGAGTTCATGACGAACGGCCCGCTCAATAGGCT
GCTCTTCGACAACTCCAGGCCACACTGGAATACCCGGGTCCATATTGCCCT
GGGAGTGGCAAGGGGACTGCTCTACCTGCACGACGAGTGCTCTAAGCAGA
TCATCCATTGCGATATCAAGCCTCAGAACATCCTGCTCGACGATAATCTCGT
GGCCAAGATCTCAGATTTCGGCCTGGCGAAGCTGCTCCTGACCAACCAGA
CACGCACGAATACCGGCATCCGCGGCACAAGGGGCTACGTCGCACCTGAG
TGGTTCAAGAACATCGGCATCTCTACCAAGGTGGACGTCTACTCATTCGGC
GTCATCCTCCTGGAGCTCGTGTGCTGCCGCAGGAATGTCGAGCTGGAGGTG
GTCGATGAGGAGCAGACCATCGTGACATACTGGGCCAATGACTGCTACAGG
TCTGGAAGGATTGATCTCCTGGTGGAGGGCGACGATGAGGCCATCTACAAT
ATCAAGAAGGTCGAGAGGTTCGTGACTGTGGCCCTGTGGTGCCTCCAGGA
GGACCCGTCTATGCGGCCAAACATGCTCAAGGTGACACAGATGCTGGATGG
AGCCGTGGCAATCCCATCCCCGCCGGACCCGTGCAGCTTCATCAGCTCTCT
CCCGTAG
