# Supplementary material for: Identification, Cloning, and Characterization of Two Acupuncture-Injury-Inducing Promoters in Rice
Source: Int J Mol Sci. 2024 Sep 30;25(19):10564. doi: 10.3390/ijms251910564 (PMC11476359; doi:10.3390/ijms251910564)
Supplement: Supplementary file 1 [file ijms-25-10564-s001.zip › Supplementary S4 Identification criteria for resistance of brown planthopper.pdf]

Identification method of resistance to brown planthoppers at the seedling stage: The material to be identified was sown in the culture box. When the rice material grew to the two-leaf period, 7 ~ 8 brown planthopper nymphs with the age of 2 ~ 3 days old were added to each plant. When more than 90% of control ZH11-WT died, each plant was scored and photographed according to the established grading criteria of 1 to 9. The final mean value of each line was the resistance level of the material to be identified.

Resistance score	Resistance level	Rice state
0	immunity	Healthy plant
1	high resistance	Slightly damaged
3	resistance	Most plants presenting the first and second leaves were partially yellowing
5	moderate resistance	Leaves showed typically yellowing, and nearly half of plants shriveled or died
7	susceptible	More than half of the plants died, and the rest were severely dwarfed
9	highly susceptible	All plants were dead
